# A High-Speed SSVEP-Based BCI Using Dry EEG Electrodes

**DOI:** 10.1038/s41598-018-32283-8

**Published:** 2018-10-02

**Authors:** Xiao Xing, Yijun Wang, Weihua Pei, Xuhong Guo, Zhiduo Liu, Fei Wang, Gege Ming, Hongze Zhao, Qiang Gui, Hongda Chen

**Affiliations:** 1 The State Key Laboratory on Integrated Optoelectronics, Institute of Semiconductors, Chinese Academy of Sciences, Beijing, 100083 China; 2 The University of Chinese Academy of Sciences, Beijing, 100049 China; 3 CAS Center for Excellence in Brain Science and Intelligence Technology, Shanghai, China

## Abstract

A high-speed steady-state visual evoked potentials (SSVEP)-based brain-computer interface (BCI) system using dry EEG electrodes was demonstrated in this study. The dry electrode was fabricated in our laboratory. It was designed as claw-like structure with a diameter of 14 mm, featuring 8 small fingers of 6 mm length and 2 mm diameter. The structure and elasticity can help the fingers pass through the hair and contact the scalp when the electrode is placed on head. The electrode was capable of recording spontaneous EEG and evoked brain activities such as SSVEP with high signal-to-noise ratio. This study implemented a twelve-class SSVEP-based BCI system with eight electrodes embedded in a headband. Subjects also completed a comfort level questionnaire with the dry electrodes. Using a preprocessing algorithm of filter bank analysis (FBA) and a classification algorithm based on task-related component analysis (TRCA), the average classification accuracy of eleven participants was 93.2% using 1-second-long SSVEPs, leading to an average information transfer rate (ITR) of 92.35 bits/min. All subjects did not report obvious discomfort with the dry electrodes. This result represented the highest communication speed in the dry-electrode based BCI systems. The proposed system could provide a comfortable user experience and a stable control method for developing practical BCIs.

## Introduction

The brain-computer interface (BCI) technique provides a direct communication pathway between the brain and the external world by translating signals from brain activities into machine codes or commands^1^. It has a wide application potential in our daily life. For example, electroencephalogram (EEG) has been used to control different types of external devices, such as a computer cursor, cellphone, home equipment or a wheelchair^2–6^. These EEG-based BCIs have provided new communication methods for either disabled or healthy people.

BCI has a variety of paradigms including P300, motor imagery and steady-state visual evoked potentials (SSVEPs). SSVEP has been widely used in BCI due to high information transfer rate (ITR), little training and high reliability^7–10^. For example, in ref.^11^, Chen *et al*. obtained an ITR of 267 bits/min, the highest ITR to date, in a 40-target SSVEP BCI system. However, this result was achieved by gel-based wet electrodes. Gel needs to be injected to the electrode before system use. During recording, the gel tends to dry out over time. After recording, the user needs to wash out the gel in the user’s hair and the EEG cap also needs to be cleaned. These procedures are time consuming and result in an uncomfortable user experience. Despite the high ITR of an SSVEP BCI, there is a certain distance between the BCI equipment and the requirement for practical applications. Major challenges include electrodes and devices for convenient EEG acquisition, and highly efficient artifact removal techniques.

In order to simplify the preparation and process of wet electrodes, many types of dry-contact electrodes have been developed. They can be classified as micro-needle^12,13^, tips^14,15^, spring pin^16^, and soft conductive polymer^17–19^ electrodes. Those types of electrodes reach the advantage of low contact impedance, high signal quality, and comfortable user experience. However, there are few reports about dry electrodes used in BCI systems and the results of ITRs still showed large room for improvement. Table 1 summarizes the literatures of dry electrode used in BCI in recent years. Many groups have made efforts to improve the performance of the dry-electrode based BCI system. For example, Mihajlovic *et al*.^20^ used 8 metal pin-based dry electrodes to acquire SSVEPs, which could identify 4 targets with accuracy of 63% and ITR of 23 bits/min. Chi *et al*.^14^ designed a mobile BCI system, in which the data acquisition module was a wireless portable box and the signal processing was accomplished on a cellphone. The system could identify 12 targets from 3 spring-loaded-pin dry electrodes. Unlike traditional Fast Fourier Transform (FFT) algorithm to extract the features of SSVEPs, they used canonical correlation analysis (CCA) method to match the templates of sin/cos waveform. The average accuracy and ITR were 89% and 26.5 bits/min. Luo *et al*.^21^ proposed a novel stimulus-locked inter-trace correlation method for 4-target SSVEP classification using EEG time-locked to stimulus onsets, which only needed one spring-loaded-pin dry electrode. The accuracy and peak ITR reached 75.8% and 34.3 bits/min. In ref.^22^, Lo *et al*. designed a wireless control system using a non-contact metal plate electrode and an FFT-based algorithm. The accuracy and the best ITR of the system were 91.1% and 38.28 bits/min in the classification of 12 targets. In addition, Martin Spuler *et al*.^23^ used 15 g.Sahara electrodes (g.tec, Graz) to acquire code-modulated VEP, which could identify 32 targets with average accuracy of 76% and average ITR of 46 bits/min, suggesting peak ITRs over 100 bit/min are possible using dry EEG electrodes.

**Table 1:** The performance of dry-electrode based BCI in recent years.

Literature	Electrode type	Paradigm	Algorithm	System calibration	Subject numbers	Health status	Electrode numbers	Target numbers	Accuracy (%)	ITR (bits/min)
1^34^	Metal pin	P300	Averaging	Yes	4	healthy	8	80	85	NA
2^34^	Solid-gel electrode	P300	Averaging	Yes	4	healthy	8	80	86.7	NA
3^31^	Gold pin (g.tec)	P300	Averaging	Yes	23	healthy	8	50	90.4	NA
4^19^	Bristle shape	MI	CSP	Yes	8	healthy	5	2	78.8 ± 13.6	NA
5^35^	Comb-shaped pin	MI	PSD	Yes	10	healthy	3	2	81.3	6.6 ± 10.8
6^36^	Metal pin	MI	CSP	Yes	5	healthy	6	2	86.8	9.6
7^24^	Non-Contact	SSVEP	CCA	No	3	healthy	3	12	83 ± 0.2	14.5 ± 6.85
8^37^	Gold cup	SSVEP	FFT	No	3	healthy	1	2	96	18.23
9^14^	Spring-loaded pin	SSVEP	CCA	No	10	healthy	3	12	89 ± 7	26.5 ± 4.2
10^20^	Metal pin	SSVEP	PSD	No	6	healthy	8	4	63	23
11^21^	Spring-loaded pin	SSVEP	Stimulus locked inter-trace correlation	No	14	healthy	1	4	75.8	34.3
12^22^	Non-Contact	SSVEP	FFT	No	10	patients	1	12	91.1	38.28
13^23^	g.Sahara electrodes	c-VEP	CCA	Yes	12	healthy	15	32	76	46
**This work**	**Claw shape**	**SSVEP**	**TRCA**	**Yes**	**11**	**healthy**	**8**	**12**	**93**.**2**	**92**.**35**

Although dry electrodes show advantages of easy preparation and no need for cleaning after use, the signal quality acquired by dry electrodes is generally lower than that of wet electrodes. The quality of EEG is the key to the communication speed of BCI. Data with lower signal-to-noise ratio (SNR) require longer lengths for accurate target identification, leading to decreased ITRs. To compensate for the SNR decrease in dry electrodes, more efficient target identification algorithms can be employed to improve the speed and accuracy in SSVEP detection. Currently, for EEG recorded with dry electrodes, the efficacy of the individual template based SSVEP detection method still remains unknown. This study aimed to explore the speed limit of an SSVEP BCI using dry electrodes. Dry electrodes and the state-of-the-art algorithm were combined to develop a high-speed SSVEP BCI with improved feasibility and practicality. Firstly, we designed a claw-like flexible dry electrode to replace the traditional wet electrode or rigid dry electrodes. It can improve user comfort and acquire good signal quality. Secondly, we adopted an efficient SSVEP detection algorithm based on task-related component analysis (TRCA). The system only required 1-second-long SSVEP data to distinguish 12 targets with high accuracy. By combining these two factors, the proposed BCI system achieved high accuracy (93.2%) and ITR (92.35 bits/min).We hope the speed improvement of the dry-electrode based SSVEP BCI can pave the road for applications in both patients with motor disabilities and healthy people. For example, patients with Amyotrophic Lateral Sclerosis (ALS) can communicate with others using the SSVEP based BCI system.

## Method

### Dry Electrodes

**Figure 1:**
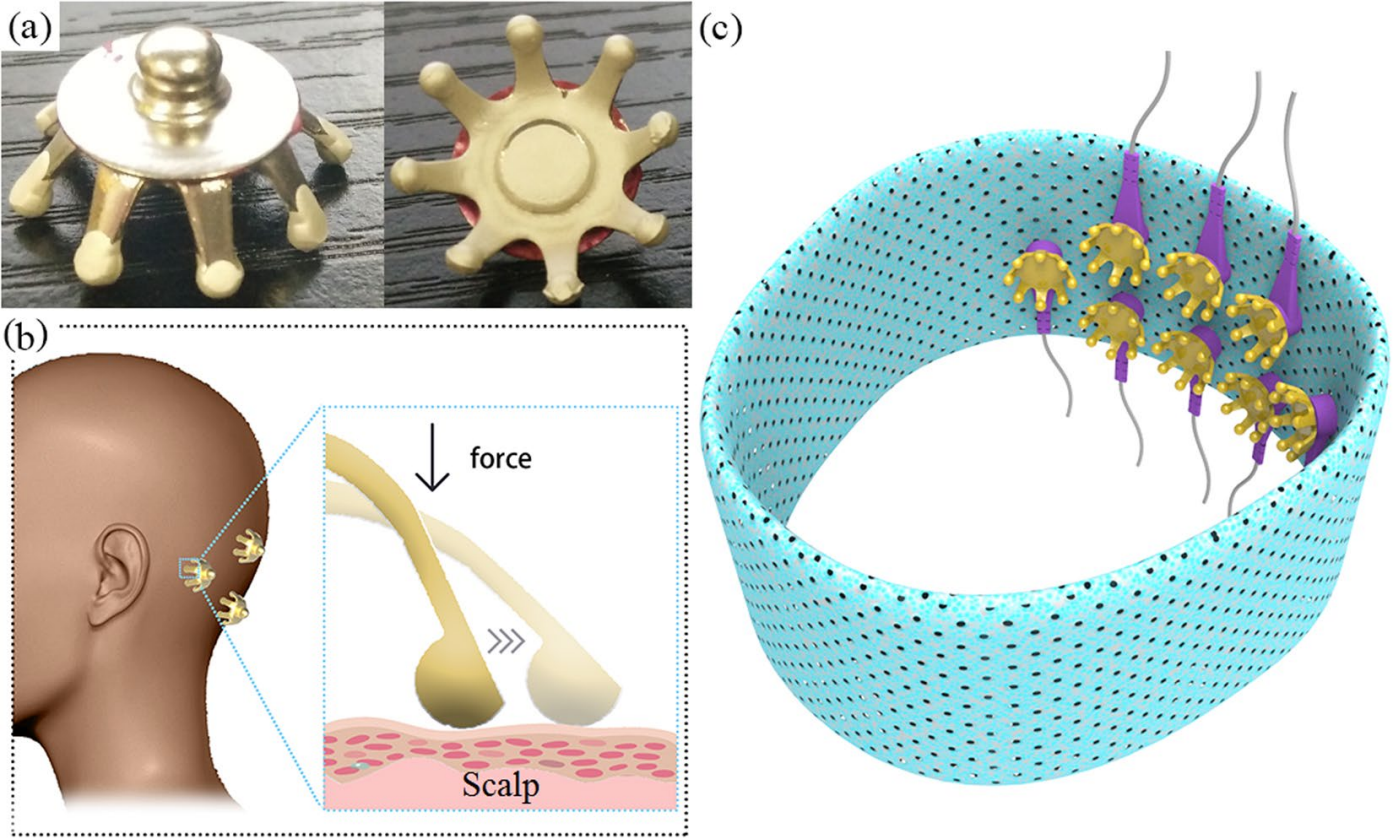
(**a**)The dry claw electrode, (**b**) the illustration of electrodes on the scalp, and (**c**) the soft headband with dry claw electrodes.

The electrode used in this study was shown in Fig. 1(a). It was designed as a claw-like structure, which was similar to the design of flexible dry electrodes in^24,25^, with a diameter of 14 mm, consisting of 8 small fingers, each showing 6 mm in length and 2 mm in diameter. Thermoplastic polyurethanes (TPU), a polymer elastomer was chosen to fabricate the electrode. The shape of the electrode was manufactured by molding. The surface of the electrode was coated with conductive ink of Ag for conductivity, and the tips of electrode were coated with conductive ink of Ag/AgCl mixture to improve electrochemical performance. The structure and material characteristic made it light and flexible to wear. The electrode is capable to go through the hair and make good contact to the scalp. As shown in Fig. 1(b), considering the effect of contact area on contact impedance, the tips of claw-like dry electrode were designed to be hemispherical. The hemispherical shape helps maintain contact area at a certain range of pressure. The shape and the elastic TPU improve comfort level compared with dry electrodes made of stiff material. As shown in Fig. 1(c), the material of soft headband was elasticized fabric. Soft and elastic headband can help to maintain a stable connection between the electrodes and the scalp.

### Experiment

This study designed an SSVEP-based BCI experiment to test the dry-electrode based BCI system. During the experiment, eight dry claw electrodes were placed at a soft headband (Fig. 1(c)). The size of headband was 18 cm × 9 cm. It could acquire the EEG signals from occipital and parietal areas (PO5, PO3, POz, PO4, PO6, O1, Oz and O2), where the SSVEPs show maximal amplitudes and SNRs^7,11^, referring to the international 10–10 system. The reference and ground electrodes were placed at the forehead using commercial hydrogel skin electrodes, which can ensure good and stable contact. The signals were recorded by a Neuroscan Synamps2 system at a sampling rate of 1000 Hz. After the headband was put on and the dry electrodes were connected to the Neuroscan System, impedance of the electrodes were tested and displayed. The impedance of each the electrode can be adjusted in two minutes to ensure the largest impedance lower than 50 KΩ. If the impedances were still lower than this value after the experiment, we believed the contact was stable. Since the frequencies of the SSVEP components are generally below 90 Hz^7^, in online analysis, data were down-sampled to 250 Hz to reduce computational cost. For the comparison purpose, after subjects completed the dry electrodes-based BCI experiment, they were also asked to use the Ag/AgCl wet electrodes to complete the experiment in the same way. The test order of dry and wet electrodes was randomized. To avoid the interference of visual fatigue, the test interval between dry and wet electrodes was 4 hours. The subjects cleaned hair after the test of wet electrodes.

**Figure 2:**
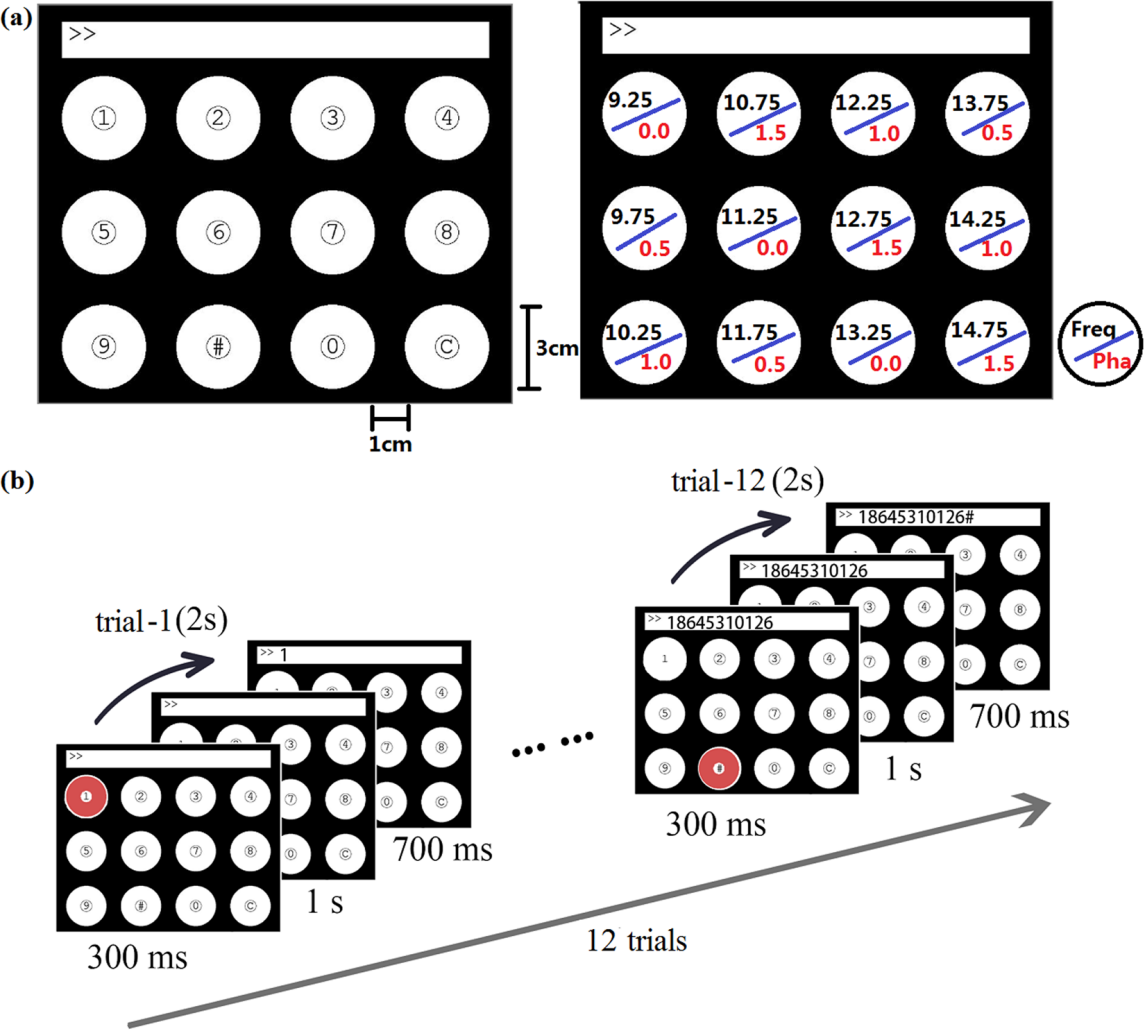
(**a**) Visual stimulus layout together with frequency and phase values for encoding the stimuli. (**b**) The timeline of a trial in the experiment.

The visual stimulator of the BCI consisted of 12 flickering stimuli rendered on a PC monitor with a 60 Hz refresh rate. This study used the sampled sinusoidal stimulation method^9^ to present the frequency-phase coded stimuli, which were proved accurately in previous studies^11,26^. As shown in Fig. 2(a), the stimuli were arranged in a 3 × 4 matrix as a virtual keypad of a phone^26^, and tagged with different frequencies and phases. The frequency range was selected from 9.25 Hz to 14.75 Hz with an interval of 0.5 Hz. The phase values started from 0 and the phase interval was 0.5π. The stimulus program was developed under MATLAB (MathWorks, Inc.) using the Psychphysics Toolbox Version 3^27^.

Eleven subjects (4 females, mean age: 25 years old) were recruited from Chinese Academy of Sciences (CAS) and took part in the experiment. All of them had normal or corrected to normal vision and had no history of central nervous system abnormalities. This study was approved by CAS’s Institutional Review Board. All experimental protocols were conducted in accordance with CAS ethical guidelines and informed consent was obtained from all participants. During the experiment, subjects seated in a comfortable chair 60 cm in front of the screen in a normally lit room. The experiment was divided into a training stage and a testing stage. The training data were used to design spatial filters and EEG templates, which were used for target detection in the testing stage.

The training stage consisted of 10 blocks. Each block contained 12 trials corresponding to 12 targets presented in a random order. As shown in Fig. 2(b), each trial started with a visual cue indicating a target stimulus. The cue appeared for 0.3 s on the screen. Subjects were asked to shift their gaze to the target as soon as possible within the cue duration. Then the stimuli started to flicker for 1 s. After stimulus offset, the screen was blank for 0.7 s before the next trial began. Therefore, each trial lasted for 2 s. There was a rest to avoid visual fatigue between two consecutive blocks.

The testing stage consisted of 5 blocks, each including 12 trials. The cue time, stimulus time and rest time were as the same as the training stage. A short beep was sounded after a target was correctly identified by the online analysis program. At the same time, the target character was typed in the text input field on the top of the screen.

### Identification Algorithm

**Figure 3:**
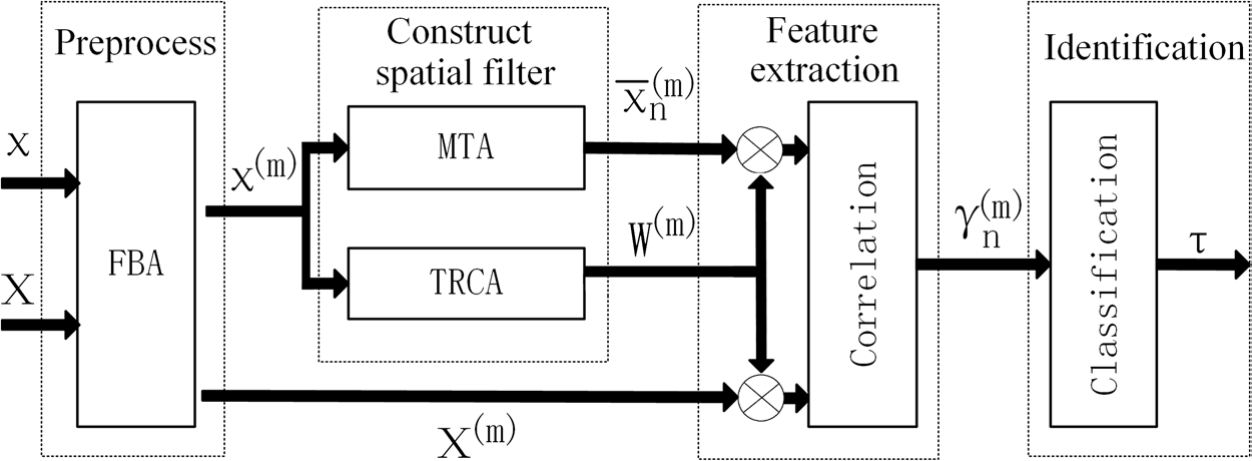
The flowchart of the TRCA-based identification method.

Figure 3 showed the flowchart of the TRCA-based target identification method^28^. It was mainly divided into four steps: preprocessing, construction of spatial filters, feature extraction and identification. Individual calibration data and single-trial test data for the *n*-th stimulus are denoted by ${{\rm{\chi }}}_{{\rm{njkh}}}\in {{\rm{R}}}^{{\rm{Nf}}\times {\rm{Nc}}\times {\rm{Ns}}\times {\rm{Nt}}}\,\,$and ${\rm{X}}\in {{\rm{R}}}^{{\rm{Nc}}\times {\rm{Ns}}}$ respectively. Here, *n* indicates the stimulus index, *N*_*f*_ is the number of stimuli, *j* indicates the channel index, *N*_*c*_ is the number of channels, *k* indicates the index of sample points, *N*_*s*_ is the number of sampling points in each trial, *h* indicates the index of training trials, and *N*_*t*_ is the number of training trials.

First, the training data χ are processed by filter bank analysis (FBA), where the SSVEPs are decomposed into *m* sub-band components. In filter bank analysis, the lower and upper cut-off frequencies of the *m*-th sub-band were set to *m* × 8 Hz and 90 Hz, respectively. According to^7^, we chose *m* = 5. After applying *m* zero-phase Chebyshev Type I Infinite impulse response (IIR) filters, the training data and test data are denoted as χ^(m)^ and X^(m)^. Zero-phase forward and reverse filtering was implemented using the filtfilt () function in MATLAB.

Second, spatial filters for the *n*-th stimuli ${{\rm{W}}}_{{\rm{n}}}^{({\rm{m}})}$ are obtained through TRCA from individual calibration data X^(m)^ as Equation (1):

1 $$\hat{w}={{\rm{\arg }}}_{w}\,{\rm{\max }}\,\frac{{w}^{T}Sw}{{w}^{T}Qw}$$

The optimal coefficient vector is the first eigenvector of the matrix Q^−1^S^29^, where Q is the covariance of concatenated matrix of all training trials across the stimuli, and S is the correlation coefficient matrix of the *n*-th stimulus between all training trials. By integrating all *N*_*f*_ coefficient vectors, we can obtain ensemble spatial filters W^(m)^.

Meanwhile, data for the multiple training trials are averaged (MTA) to obtain the individual template ${\overline{{{\rm{\chi }}}_{{\rm{n}}}}}^{({\rm{m}})}$.

Third, individual template ${\overline{{{\rm{\chi }}}_{{\rm{n}}}}}^{({\rm{m}})}$ and test data X^(m)^are multiplied with spatial filters W^(m)^respectively, and then the Pearson’s correlation coefficient ${{\rm{\gamma }}}_{{\rm{n}}}^{({\rm{m}})}$ between them can be calculated.

Last, the final features ρ_n_ can be obtained by merging the correlation coefficients ${{\rm{\gamma }}}_{{\rm{n}}}^{({\rm{m}})}$ as Equation (2), and the target class τ can be identified as Equation (3):

2 $${{\rm{\rho }}}_{{\rm{n}}}=\sum _{m=1}^{{N}_{m}}a(m)\cdot {({\gamma }_{n}^{(m)})}^{2}$$

3 $${\rm{\tau }}={{\rm{\arg }}}_{n}{\max {\rm{\rho }}}_{n}\,n=1,2\ldots {N}_{f}$$

where, N_*m*_ is the total number of sub-bands, and ${\rm{a}}(m)={m}^{-1.25}+0.25$ according to^7^.

### Performance Evaluation

#### Impedance of the electrode

The impedance property of dry electrode was evaluated using electrochemical impedance spectroscopy (EIS). Firstly, in order to make a controlled and consistent testing environment, the data were measured by an electrochemical workstation (CHI 660D, China) and tested in 0.9% NaCl solution. The proposed dry electrode was set as the measurement electrode and standard Pt electrodes were set as the reference and ground electrodes. Sinusoidal AC signals with voltage amplitude of 10 mV, frequencies from 0.1 Hz to 1000 Hz, were applied to measure the EIS. For comparison purposes, commercial Ag/AgCl wet electrodes were measured using the same method. The experiment was repeated three times. Secondly, the impedance of the dry electrode was tested on volunteers’ head^30^. Two dry electrodes, one as the working electrode and the other as the reference electrode, were placed 3 cm away on the occipital region. An elastic headband was used to compress them on the head of subjects. EIS of the dry electrode was tested with the same condition as that in the wet environment. The value would be divided by 2 to get the average impedance of one dry electrode. For comparison, commercial Ag/AgCl wet electrodes were measured with commercial gel (Compumedics Neuromedical Supplies^TM^) on head using the same method. The test was performed on five subjects.

#### Signal quality of the electrode

The signal quality of the dry electrode was evaluated by calculating the correlation coefficient and comparing the SNR of SSVEP components between the dry and wet electrodes.

The signal was collected from the dry electrode and Ag/AgCl wet electrode at the same time. They were placed at the area near the Oz position, and the distance was 2 cm apart. The reference and ground were wet electrodes on the ear lobes (A1 and A2 position, respectively). The signals were recorded by a Neruoscan Synamps2 system at a sampling rate of 1000 Hz. The test consisted of 5 trials. In one trial, there were 4 flickers rendered on a monitor in turn and the frequencies were 10 Hz, 12 Hz, 15 Hz, and 20 Hz. Each stimulus lasted for 10 s followed by a rest for 5 s.

Before analyzing the data, data epochs of 10 s data length were extracted according to the event triggers generated by the stimulus program. All data epochs were first down-sampled to 250 Hz and then filtered with a 50 Hz notch filter to remove the power line noise.

The correlation coefficient was calculated as follows:

4 $${\rm{R}}={\rm{cov}}(x1,x2)/\sqrt{{\rm{cov}}(x1,x1)\,{\rm{cov}}(x2,x2)}\,$$

where R symbolized the correlation coefficient, cov(.) represented covariance, and *x*1 and *x*2 represented filtered signals recorded by the dry and wet electrodes respectively.

The SNR was calculated as follows:

5 $${\rm{SNR}}=20\ast {\mathrm{log}}_{10}\frac{y(f)}{y(f-1)+y(f+1)}\,$$

The amplitude spectrum *y*(*f*) was calculated by FFT. SNR in decibels (dB) was defined as the ratio of *y*(*f*) to the mean value of the 2 neighboring frequencies (i.e. one frequency on each side).

#### Performance of the system

The performance of the BCI system was evaluated using the classification accuracy and ITR. The ITR was calculated as follows:

6 $${\rm{ITR}}=\frac{60}{T}[{\mathrm{log}}_{2}N+P{\mathrm{log}}_{2}P+(1-P)\,{\mathrm{log}}_{2}\frac{1-P}{N-1}))]$$

where P was the accuracy, T was the average time for a command (T = 2 s), and N was the number of commands (N = 12).

#### Comfort level assessment

The comfort level of the electrodes was assessed in the form of a survey. All subjects were asked to report the comfort level of the proposed electrode and a commercial dry electrode (purchased from Wearable Sensing Company, its pin is fabricated from a stiff metal material) after wearing the electrodes longer than 1 h. Comfort levels are divided into four grades: (1) Comfort with gentle sense of pressure but no pain, (2) Slight pain, (3) Pain but acceptable, and (4) Obvious pain with discomfort^19,31,32^.

## Results

### Dry Electrode Performance

#### Impedance of the electrode

**Figure 4:**
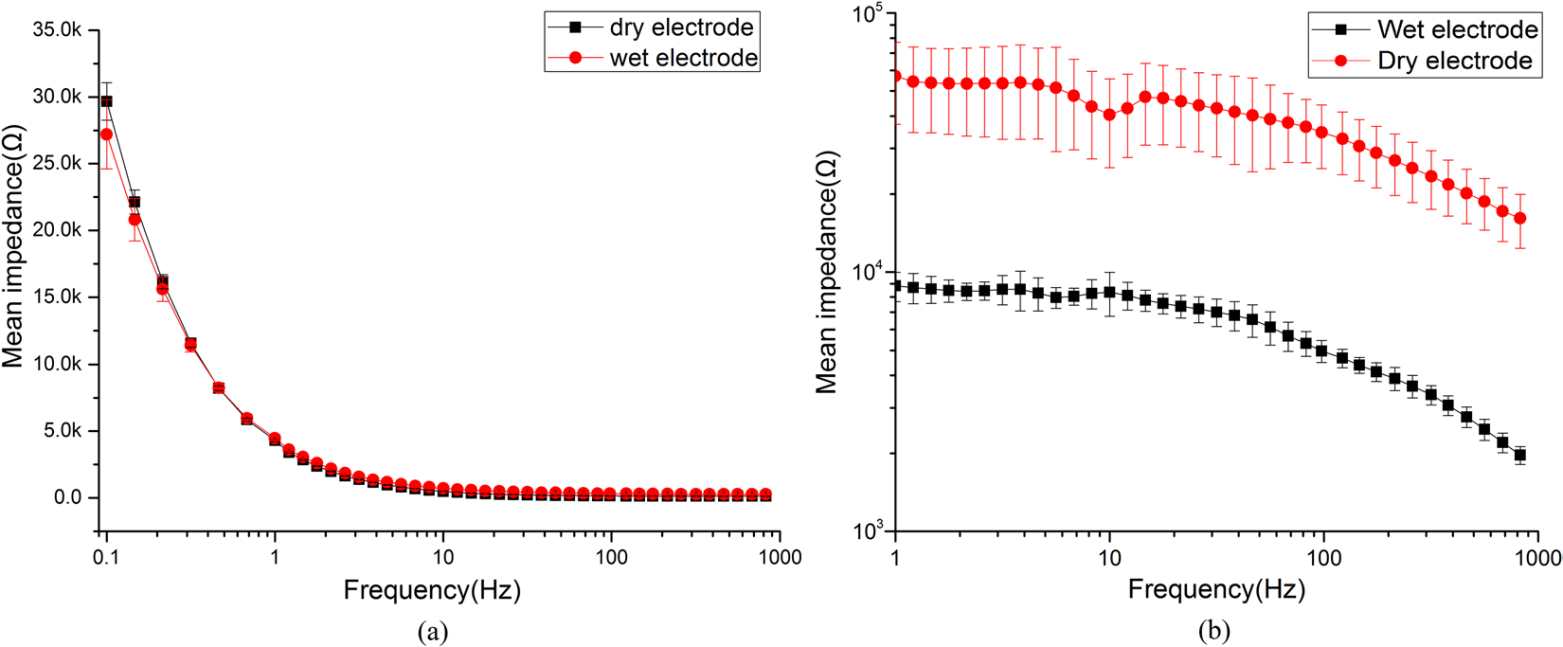
(**a**) The amplitude of EIS from dry electrode and wet electrode in 0.9% NaCl solution. (**b**) The amplitude of EIS from dry electrode and wet electrode on the scalp.

Results of EIS in wet condition were presented in Fig. 4(a). The impedance will decrease as the frequency increase. At the frequency around 10 Hz, the average impedance of dry electrode and wet electrode were 1.52 ± 1.54 KΩ and 1.72 ± 1.53 KΩ respectively. There was no clear difference of the mean impedance. It means that the proposed dry electrodes will have similar impedance property to that of the commercial wet electrodes if they can contact with the scalp with large enough area. However, without the help of gel, the effective contact area of the dry electrode is very small. As shown in Fig. 4(b), the dry-contact test result shows that the average impedance is 38.6 KΩ ± 9.5 KΩ (@10 Hz) on the scalp. Compare with the impedance of wet electrodes, 8.3 KΩ ± 1.67 KΩ (@10 Hz), the value is 4–5 fold higher.

#### EEG signal quality

**Figure 5:**
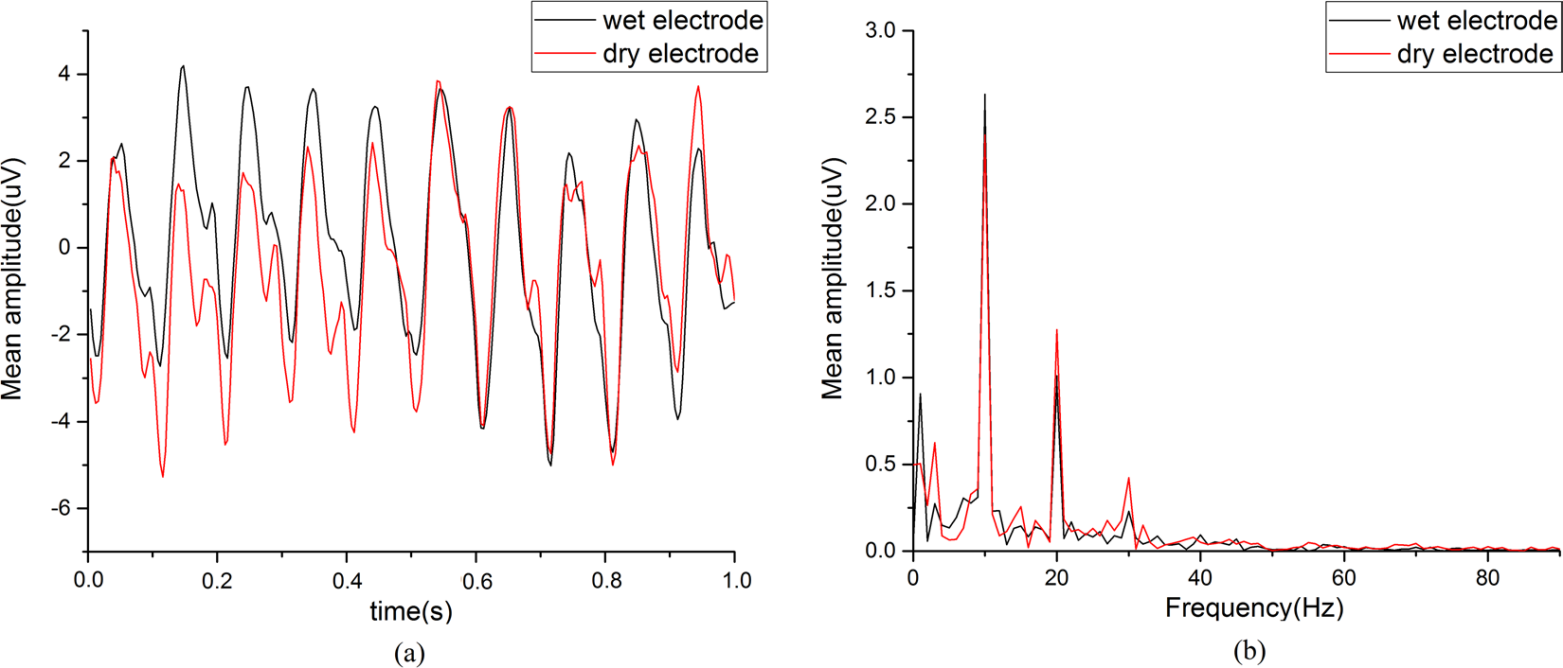
(**a**) The averaged temporal waveform and (**b**) amplitude spectrum of 10 Hz SSVEP from all subjects corresponding to dry electrode (black) and wet electrode (red).

Figure 5 showed the averaged temporal waveform and amplitude spectrum of SSVEP at 10 Hz collected from the dry electrode and wet electrode from all subject. Both electrodes could acquire clear SSVEPs that exhibited peak frequencies at 10 Hz and 20 Hz. The temporal waveforms for the two electrodes were highly correlated to each other (R = 0.83).

Table 2 showed the SNR and correlation coefficients from the two electrodes at 4 stimuli for all subjects. The average SNR across frequencies for the dry electrode and the wet electrode were 12.83 ± 2.85 dB and 14.1 ± 3.24 dB respectively. The average correlation coefficient between dry electrode and wet electrode was 0.66 ± 0.02.

**Table 2:** The quality of SSVEPs from dry electrode and wet electrode.

Stimulus (Hz)	SNR (dB)		R
	Dry electrode	Wet electrode	Dry vs Wet
10	14.33 ± 2.21	17.02 ± 1.14	0.69 ± 0.03
12	14.54 ± 2.44	16.34 ± 1.87	0.66 ± 0.02
15	12.17 ± 1.92	11.12 ± 2.59	0.63 ± 0.03
20	10.29 ± 2.98	11.94 ± 2.1	0.64 ± 0.03
Average	12.83 ± 2.85	14.1 ± 3.24	0.66 ± 0.02

R is correlation coefficient.

### BCI Performance

The results of the online BCI experiment were shown in Table 3. For the wet electrode, the average accuracy and ITR of all 11 subjects was 97.35 ± 4.33% and 101.28 ± 9.46 bits/min. Compared to the wet electrode, the average accuracy of dry electrode was 93.2 ± 5.74% and ITR was 92.35 ± 12.08 bits/min. Paired t-tests indicated significant difference of classification accuracy (p < 0.05) and ITR (p < 0.05) between the two type of electrodes. Four (S3, S5, S6, and S8) and two subjects (S3, S5) obtained 100% accuracy using the wet electrode and the dry electrode respectively. Compared with the TRCA algorithm, offline analysis using the CCA algorithm obtained decreased accuracy and ITR. The difference of accuracy between CCA and TRCA was significant for both wet (89.9% vs 97.35%, p < 0.01) and dry (74.82% vs 93.2%, p < 0.01) electrodes. These results indicate that the TRCA algorithm plays a key role in achieving high ITR with the dry-electrode based SSVEP BCI.

**Table 3:** The performance of BCI from dry and wet electrodes.

Subject	Accuracy (%) based on CCA		Accuracy (%) based on TRCA		ITR (bits/min) based on CCA		ITR (bits/min) based on TRCA	
	Dry electrode	Wet electrode	Dry electrode	Wet electrode	Dry electrode	Wet electrode	Dry electrode	Wet electrode
S1	81.67	95	98.33	98.33	67.91	93.76	103.29	103.29
S2	77.50	87.5	94.44	95.84	61.12	78.27	95.24	96.99
S3	84.17	100	100	100	72.21	107.49	107.49	107.49
S4	75.00	88.9	93.33	96.67	57.26	80.94	91.74	99.09
S5	83.33	100	100	100	70.74	107.49	107.49	107.49
S6	72.22	95.83	91.67	100	53.14	95.72	89.05	107.49
S7	66.67	87.22	85	98.33	45.41	77.74	77.36	103.29
S8	80.56	85.00	98.33	100	66.06	73.68	103.29	107.49
S9	63.33	75.00	83.33	85	41.05	57.26	73.03	79.8
S10	69.44	83.33	90.8	98.3	49.19	70.74	84.71	103.29
S11	69.17	77.20	90	98.3	48.81	60.65	83.1	103.29
Average	74.82	89.9	93.2	97.35	57.54	82.15	92.35	101.28

### Comfort Level of Electrode

Most participants in this study had previous experience of traditional gel-based wet electrodes and different types of dry electrodes. All of them thought the pin-type dry electrode caused stronger feeling of oppression than the claw dry electrode. For the claw dry electrode, four subjects chose level 1, six subjects chose level 2, and one subject chose level 3. In contrast, for the pin-type electrode, one subject chose level 1, four subjects chose level 2, and others chose level 3. Compared with the pin-type electrode, the deformation of the claw electrode relieves the skin pressure to a certain extent.

## Discussion

This study obtained the highest ITR (92.35 bits/min) reported in the dry-electrode based BCIs (see Table 1). Specifically, the ITRs of the calibration-free SSVEP BCI systems ranged from 14.5–38.28 bits/min. The 32-target code-modulated VEP system using the individual template-based CCA algorithm^23^ obtained an ITR of 46 bits/min. The proposed system benefited from the higher classification accuracy and shorter stimulation duration, which were contributed by the self-fabricated dry electrodes and the adopted TRCA-based detection algorithm. By optimizing the structure and materials, the claw-like dry electrode acquired good signal quality and provided reliable data for subsequent signal processing. The TRCA algorithm fully considered the individual differences and took individual calibration data as template, so it achieved high classification accuracy with a short data length.

The TRCA algorithm requires system calibration to obtain individual templates and spatial filters. In this study, the training procedure included 10 blocks, which lasted 4 minutes in total. The systems that do not require system calibration are more user friendly. However, as shown in Table 3, the unsupervised CCA algorithm showed a large drop (TRCA: 93.2%, CCA: 74.82%) of accuracy with the dry electrodes. This finding suggests that a much longer stimulation duration is required to obtain high accuracy using the CCA method. Alternatively, zero-training methods such as the session-to-session transfer algorithm^33^ could be an effective way to facilitate system implementation using the TRCA algorithm.

**Figure 6:**
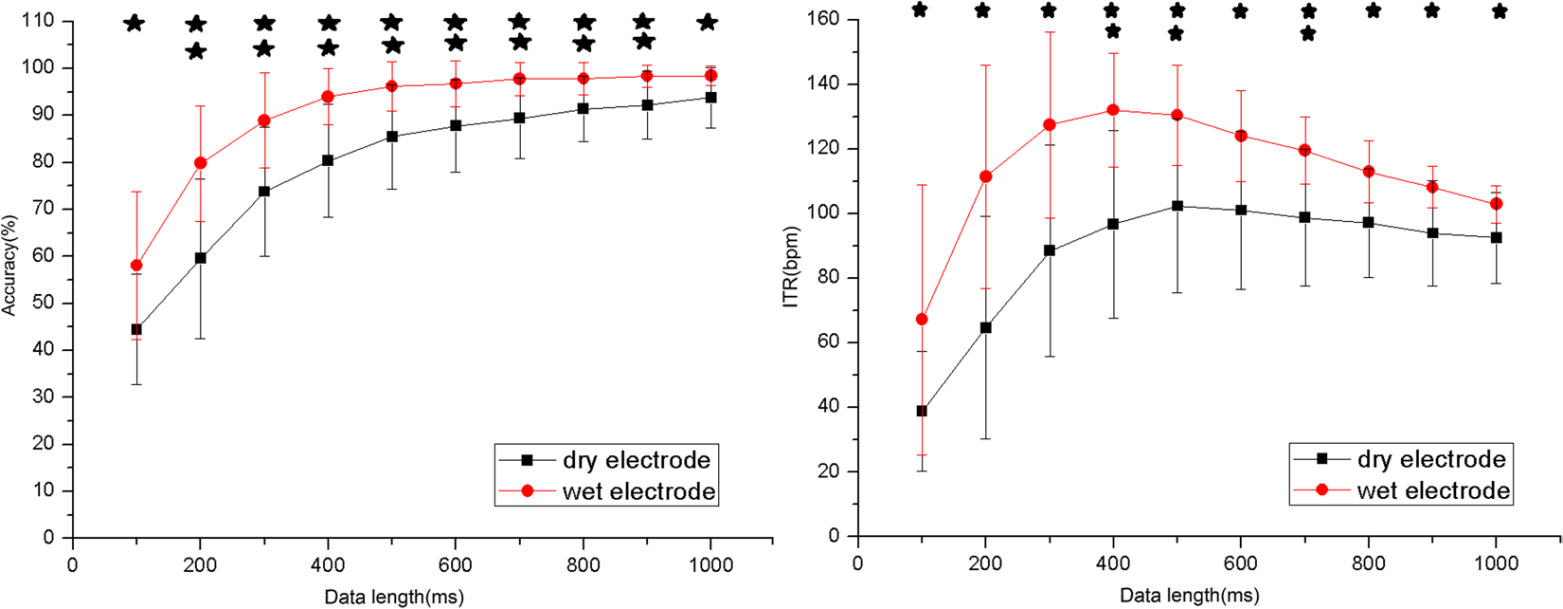
Results of average classification accuracy (**a**) and ITR (**b**) with different data lengths from dry electrode (black) and wet electrode (red). (*p < 0.05, **p < 0.01).

The online BCI system used 1 s stimulation duration towards high classification accuracy. However, the ITR can be further improved by optimizing the stimulus duration. Figure 6 showed the results of average classification accuracy and ITR across all subjects with different training data lengths from dry electrode and wet electrode. The accuracy and the ITR were estimated by a leave-one out cross validation, in which 9 blocks were used as training data and 1 block was used as test data. For the wet electrode, the highest ITR was 129.92 ± 16.56 bits/min with the data length of 400 ms and the accuracy was 93.34 ± 5.58%. For the dry electrode, the highest ITR was 102.37 ± 26.92 bits/min with the data length of 500 ms and the accuracy was 85.45 ± 11.06%. This result suggested we could obtain higher ITR by optimizing stimulus duration (e.g., 500 ms).

**Figure 7:**
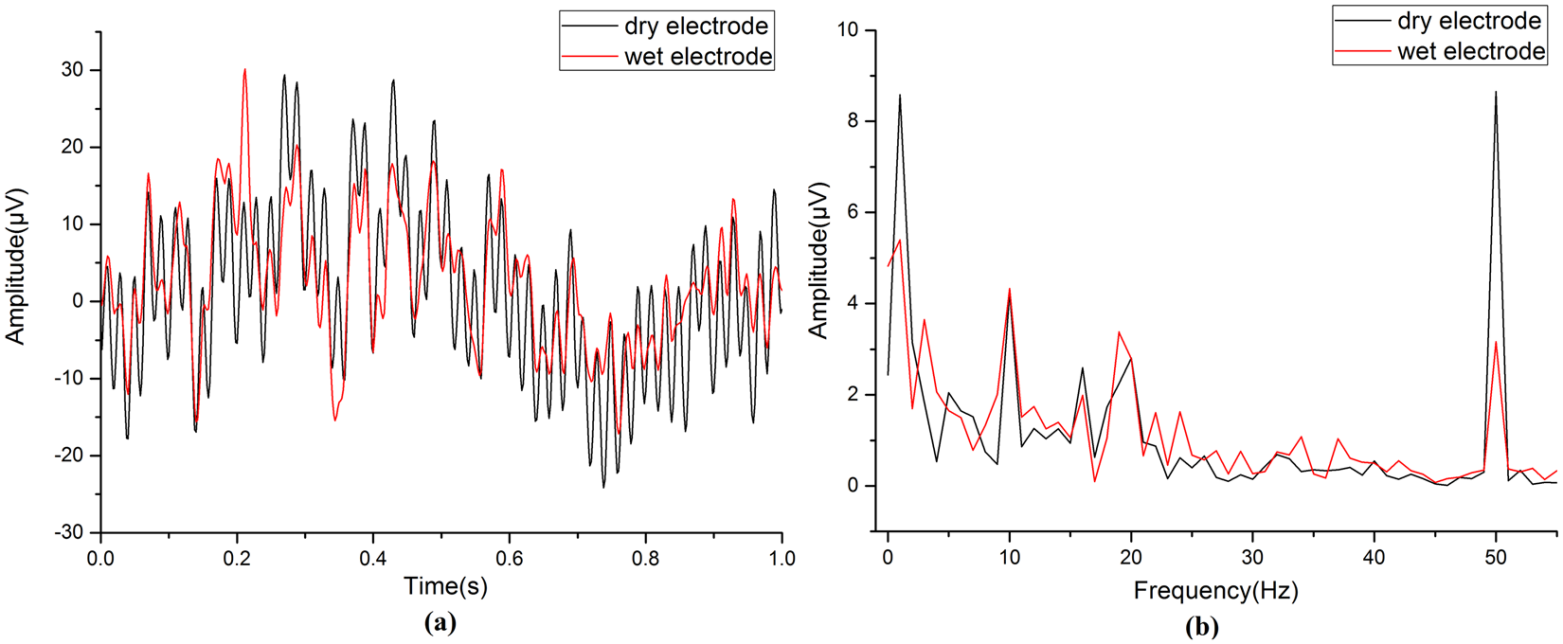
The original temporal waveform (**a**) and spectrum (**b**) of 1s-long data from dry electrode (black) and wet electrode (red).

Paired t-tests showed the accuracy and ITR of two kinds of electrodes had significant difference for all data lengths. It meant the signal quality of short data was still different between dry and wet electrodes. Figure 7(a) showed the original 10 Hz SSVEP of 1 s stimulation duration without filtering. From the amplitude spectrum in Fig. 7(b), in the signal frequency band of SSVEP (8–30 Hz), there was no large difference. But in the low frequency band (0–5 Hz) and at the power line frequency (50 Hz), the dry electrode acquired more interference than wet electrode obviously. The low frequency noise mainly comes from the unstable contact between electrode and skin. This instability will cause the change of electrode-skin electron double layer and therefore produce the noise. There are two ways to produce the power line noise. First, when the contact impedances of work electrode and reference electrode do not match, the power line interference will be amplified by acquisition circuit in the form of differential-mode signal and then mixed in the EEG signals. Second, the power line interference can directly couple into the EEG signals through the unshielded electrode. Therefore, in the future, the anti-interference ability of the dry electrode need to be improved from the following two aspects. First, the mechanical properties such as elasticity and hardness should be adjusted to make the dry electrode contact with the scalp in a stable and comfortable way. It is helpful to avoid the relative sliding of the electrode and thus reduce the low frequency noise. Second, the power line noise can be reduced by designing a shielding layer for dry electrode.

## Conclusion

This study demonstrates a high performance SSVEP-based BCI system using dry claw-like electrodes. The proposed dry electrode reduces the system preparation time. Its flexible increases the wearing comfort and improves the user experience. The adopted TRCA algorithm improves the classification accuracy and ITR of system. Both ergonomic factors and system performance factors are optimized towards practical BCI applications.

The material as well as structure of the dry electrode have been designed and adjusted elaborately. It can be wore comfortably on hair covered head area and the pins of the electrode can go easily through the hair and contact the scalp. The impedance of the electrode is stable and therefore the electrode is capable to record reliable and high quality signals for subsequent signal processing. The adopted TRCA-based identification algorithm can achieve high accuracy with short data by fully considering the individual differences of SSVEPs. By combining these two characteristics, the present dry-electrode based BCI system achieves high classification accuracy (93.2 ± 5.74%) and high ITR (92.35 ± 12.08 bits/min) using a 12-class BCI paradigm. These results demonstrate the feasibility of dry electrodes for the high-speed BCI technology. The proposed diagram could be used to implement SSVEP BCIs towards various applications for the communication and control purposes.
